# Reproductive Health in Young and Adolescent Females with Inflammatory Bowel Disease

**DOI:** 10.1007/s11894-026-01032-7

**Published:** 2026-02-13

**Authors:** Erica J. Brenner, Bianca A. Allison, Catalina Berenblum Tobi, Camilia Kamoun, Vidhya Krishnan, Hilary K. Michel

**Affiliations:** 1 Division of Pediatric Gastroenterology, Department of Pediatrics, University of North Carolina School of Medicine, Chapel Hill, NC USA; 2 Division of General Pediatrics and Adolescent Medicine, Department of Pediatrics, University of North Carolina School of Medicine, Chapel Hill, NC USA; 3 Division of Pediatrics, Boston Children’s Hospital, Boston, MA USA; 4 Division of Pediatric Endocrinology, Department of Pediatrics, University of North Carolina School of Medicine, Chapel Hill, NC USA; 5 Department of Social Medicine, University of North Carolina School of Medicine, Chapel Hill, NC USA; 6 Section of Pediatric and Adolescent Gynecology, Nationwide Children’s Hospital, Columbus, OH USA; 7 Division of Gastroenterology, Hepatology, and Nutrition, Nationwide Children’s Hospital, 700 Children’s Drive, Columbus, 43205 OH USA; 8 Department of Pediatrics, The Ohio State University College of Medicine, Columbus, OH USA

**Keywords:** Inflammatory bowel disease, Crohn’s disease, Ulcerative colitis, Reproductive health, Puberty, Contraception

## Abstract

**Purpose of Review:**

This manuscript addresses high-yield topics related to sexual and reproductive health (SRH) in adolescents and young women with inflammatory bowel disease (IBD) including puberty, menstruation, contraception, fertility, pregnancy, preventive care, and counseling strategies.

**Recent Findings:**

Adolescent and young women with IBD are at risk for delayed puberty, menstrual cycle disorders, unintended pregnancy, and cervical cancer. Active disease or a history of intra-abdominal surgery can negatively impact fertility and pregnancy outcomes.

**Summary:**

With appropriate screening, counseling and education, and shared decision making, providers can optimize both IBD and SRH outcomes. Further research in needed to specifically understand the role of pre-conception counseling in the adolescent setting, optimal management of menstrual cycle disorders in women with IBD, and the safety of newer medications during pregnancy.

## Introduction

Inflammatory bowel disease (IBD), including Crohn’s disease (CD) and ulcerative colitis (UC), is a systemic inflammatory condition affecting approximately 3 million people in the United States (US), nearly half of whom are female [1]. One quarter of patients are diagnosed before age 20, with incidence peaking between the second and fourth decades, coinciding with essential stages in pubertal development and sexual and reproductive health (SRH) [1]. Though most pediatric gastroenterology (GI) clinicians may not be SRH experts, having a general understanding of how IBD impacts puberty, menses, contraception, fertility, and pregnancy will help them to optimize whole-person care for this population. Such counseling and awareness are of particular importance given the current sociopolitical climate [2]. This review addresses high yield topics in SRH for females with IBD and provides expert and evidence-based recommendations (Table 1) and resources (Table 2) for the GI clinician. For the purposes of this review, we use the term “women” to describe individuals with female sex physiology at birth. We acknowledge that gender identity may differ from sex assigned at birth and that issues affecting gender-diverse individuals are important but outside the scope of the current review.

**Table 1:** High yield sexual and reproductive health recommendations for the IBD clinician

**Fig. 1:**
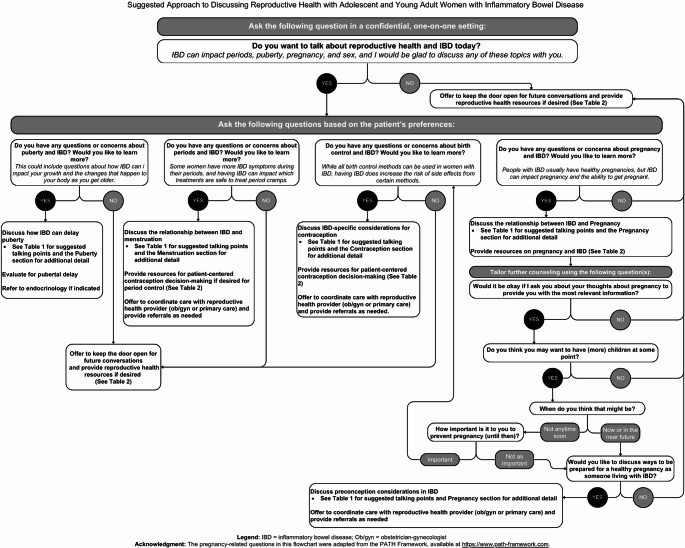
Suggested approach to discussing reproductive health with adolescent and young adult women with inflammatory bowel disease

1.Puberty • Assessment of growth parameters (height, weight, BMI) and pubertal status should be measured and tracked routinely. • The most common cause of delayed puberty is active IBD – every effort should be made to optimally control disease. • Delayed puberty (lack of breast development by chronological age of 13 years or lack of menarche within 3 years of breast development) despite a substantial period of well-controlled IBD and/or unusual clinical findings should prompt referral to pediatric endocrinology. • If obtaining a bone age, know that a bone age of less than 11 years and low ultrasensitive FSH level suggests lack of activation of the hypothalamic-pituitary-gonadal (HPG) axis, while a bone age above 13 years and low ultrasensitive FSH level is suggestive of gonadotropin deficiency. Ultrasensitive LH levels less than 0.15 U/L suggest lack of central puberty onset, while levels above 0.3 to 0.6 U/L suggest activation of the HPG axis.
2. Menstruation and Menstrual Cycle Disorders • GI symptoms are often increased around the time of menses – determining associations between symptoms and menses, treating menstrual symptoms appropriately, and assessing IBD inflammatory burden is important to improve symptom control and prevent unnecessary changes in IBD therapy. • Treatment options for menstrual cycle disorders in women with IBD include judicious use of NSAIDs, hormonal contraceptives including oral contraceptive pills, and long-acting reversible contraceptives such as the progestin-containing IUD and etonogestrel implant. • Patients with menstrual cycle disorders should be screened for associated iron deficiency anemia and managed with supplementation.
3. Contraception • There are no contraceptive methods that are absolutely contraindicated in patients with IBD, though risk of VTE should be reviewed in patients considering combined hormonal contraceptives. • Shared decision making is necessary to help patients weigh risks and benefits of contraceptive methods to best meet their needs (See Fig. 1 for guidance on having these conversations).
4. Fertility and Pregnancy • Adequately controlled disease along with medical and nutritional optimization are the best ways to support fertility and pregnancy outcomes. • No IBD medications negatively impact female fertility, and with the exception of methotrexate, long term steroids, and small molecule therapies, all can be continued in pregnancy. • In those with risk factors for infertility (i.e., history of pelvic surgery), referral to a fertility specialist is indicated at 6 months if they do not achieve spontaneous pregnancy.
5. Preventive Care • HPV vaccination is safe and recommended for all patients with IBD, especially those on immunosuppressive therapy. • Cervical cancer screening, starting at age 21 and continuing every 3 years is recommended, with consideration of shorter intervals and earlier initiation in immunosuppressed patients.
6. Counseling Approaches • Reproductive health counseling should start early, be proactive and developmentally appropriate, and initiated by healthcare teams. • Confidential, one-on-one time between provider and patient is essential to build trust, address SRH topics, and develop communication skills.

IBD Inflammatory bowel disease; BMI body mass index; FSH follicle stimulating hormone; LH luteinizing hormone; NSAIDS nonsteroidal anti-inflammatory drugs; IUD intrauterine device; VTE venous thromboembolism; HPV human papilloma virus

**Table 2:** Patient-facing resources on reproductive health in inflammatory bowel disease (IBD)*

Organization	Webpage	Description
The American Gastroenterological Association™	**My IBD Life: Intimacy and Relationships** https://myibdlife.gastro.org/scenarios/intimacy-and-relationships/	Tips on navigating intimacy and relationships for those living with IBD
Colorectal Cancer Alliance™	**Sexual Health and Intimacy with an Ostomy** https://colorectalcancer.org/resources-support/resources/living-well-colorectal-cancer/ostomy/sexual-health-ostomy	Information on sexual health and intimacy with an ostomy
The Crohn’s and Colitis Foundation™ United States	**Intimacy and Contraception in IBD** https://www.crohnscolitisfoundation.org/effects-of-ibd-on-women/intimacy-and-contraception	Information about intimacy and contraception in IBD
	**Pregnancy and IBD** https://www.crohnscolitisfoundation.org/effects-of-ibd-on-women/pregnancy-and-ibd	Information on pregnancy and IBD, including considerations about medications, disease monitoring, surgery, heredity, nutritional needs, and breastfeeding
	**Puberty and Menstruation in IBD** https://www.crohnscolitisfoundation.org/effects-of-ibd-on-women/puberty-and-menstruation	Information on puberty and menstruation in IBD
The Crohn’s and Colitis Foundation™ Canada	**Relationships and Intimacy in IBD** https://crohnsandcolitis.ca/about-crohn-s-colitis/ibd-journey/Relationships-and-Intimacy	Information about relationships and intimacy in IBD, including medications and intimacy, surgery and intimacy, and starting a new relationship
	**Fertility and Pregnancy in IBD** https://crohnsandcolitis.ca/about-crohn-s-colitis/ibd-journey/Fertility-and-Pregnancy-in-IBD	Information on pregnancy and IBD
The Crohn’s and Colitis Foundation™ United Kingdom	**Reproductive Health and Fertility in IBD** https://crohnsandcolitis.org.uk/info-support/information-about-crohns-and-colitis/all-information-about-crohns-and-colitis/living-with-crohns-or-colitis/reproductive-health-and-fertility	Information about reproductive health, including contraception, for those living with Crohn’s or colitis
	**Sex and Relationships and IBD** https://crohnsandcolitis.org.uk/info-support/information-about-crohns-and-colitis/all-information-about-crohns-and-colitis/living-with-crohns-or-colitis/sex-and-relationships	Information on possible impacts of IBD on sex and relationships
	**Pregnancy and Birth in IBD** https://www.crohnsandcolitis.org.uk/info-support/information-about-crohns-and-colitis/all-information-about-crohns-and-colitis/living-with-crohns-or-colitis/pregnancy-and-birth	Information on pregnancy and birth for people living with IBD
Generation Patient^TM^**	**Generation Patient** https://generationpatient.org/	Peer support and access to educational resources for young adults living with chronic diseases
	**Crohn’s and Colitis Young Adults Network** https://generationpatient.org/ccyan	A network that hosts community-led peer support groups for young adults (18+) with IBD
	**Roundtable Video on Sexual and Reproductive Health for Young Adults in IBD** https://www.youtube.com/watch?v=FeukcXtIFB8	Young adults with IBD discuss sexual health, reproductive health, and family planning
Girls with Guts^TM^**	**Girls with Guts** https://girlswithguts.org/	A community for women with IBD and/or ostomies
IBDMoms^TM^**	**IBDMoms** https://ibdmoms.org/	A resource run by mothers affected by IBD that provides education, resources, and social events
ImproveCareNow™ Patient Advisory Council**	**Body Image Toolkit** https://assets.nationbuilder.com/improvecarenow/pages/267/attachments/original/1645653910/2.18.22_Body_Image_Toolkit.pdf	Personal experiences of body image issues from teens and young adults living with IBD
Mother to Baby™	**Pregnancy and Breastfeeding Exposures in IBD** https://mothertobaby.org/pregnancy-breastfeeding-exposures/inflammatory-bowel-disease/	Evidence-based information on the safety of medications and other exposures during pregnancy and breastfeeding for people with IBD
University of California San Francisco	**The PIANO Research Study** https://pianostudy.org/results.php	Results from the Pregnancy in IBD and Neonatal Outcomes (PIANO) research study, a national study of women with IBD and their children
University of Chicago	**Fertility**,** Pregnancy and Sexual Function for Women with IBD** https://www.uchicagomedicine.org/conditions-services/inflammatory-bowel-disease/treatment/fertility-sex-and-pregnancy-for-women-with-crohns-and-ulcerative-colitis	Information on fertility, pregnancy, and sexual function and IBD, including the use of intestinal ultrasound in pregnancy
We Care in IBD™	**We Care in IBD** https://www.wecareinibd.com/	Information related to IBD, pregnancy, and fertility
Young Patient’s Autoimmune Research and Empowerment Alliance (AREA) ™**	**Young Patients’ Autoimmune Research and Empowerment Alliance (AREA)** https://youngpatientsarea.org/	An organization empowering young people with autoimmune conditions to have a voice in research

*The authors acknowledge that web-based resources are subject to change over time, and the content, availability, or accuracy of the listed resources may differ from those at the time of publication. Accordingly, the authors have compiled a living document of resources that will be updated periodically, available here: https://tarheels.live/brennerlab/reproductive-health-resources/. Provider-facing resources can be found in the Key References section of this publication and here: https://tarheels.live/brennerlab/reproductive-health-resources-for-providers-caring-for-patients-with-ibd/. Inclusion of these resources is provided for informational purposes only and does not constitute endorsement or verification by the authors or the publisher.**Patient-led organization

## IBD and Puberty

### Overview of Female Puberty in IBD

Female pubertal development is a complex process influenced by genetic determinants, nutritional status, and overall health status among other factors. While the exact mechanisms that lead to pubertal onset are unknown, various influences permitting and interfering with the initiation and maintenance of pubertal development have been identified. For example, insulin growth factor I and leptin, both markers of nutritional status, may promote initiation of puberty. Hunger signals (e.g., neuropeptide Y), corticosteroids, and inflammation may delay pubertal development [3, 4]. 

IBD, particularly CD, is associated with delayed puberty due to a combination of malnutrition, inflammation, and corticosteroid treatment [3–5]. Delayed puberty in a female is defined as lack of breast development by chronological or bone age of 13 years (or age 2-2.5 standard deviations above the population mean age of breast development) or lack of menarche within 3 years of breast development [3, 6]. Secondary amenorrhea or oligomenorrhea for over 1 year are also signs of abnormal pubertal progression [3]. UC is less commonly associated with delayed puberty possibly due to differences in rates of malnutrition or disease control [7–9]. A retrospective single center study of females with IBD seen between 1985 and 2016 found that 40% had delayed menarche (onset ≥ 14.5 years) [9]. Among a recent Korean cohort, 5 of 36 females older than 13 years had delayed puberty [8]. In a US-based multicenter longitudinal study of patients with CD, estimated cumulative incidence of menarche by age 14 years was only 43.6% (95% CI, 24.4–62.8%) [10]. It is unclear if delayed puberty impairs linear growth in females with IBD [8, 9]. 

### Evaluation and Management of Delayed Puberty

Evaluation of pubertal status in females with IBD should include a thorough clinical history, family history of pubertal development, and physical exam, including growth evaluation. History should not only focus on disease status but also screen for signs and symptoms of other conditions associated with abnormal pubertal development (e.g., anorexia nervosa, prolactinoma). Careful measurements of weight and height should be obtained at each visit to assess rate of weight change, body mass index (BMI), and linear growth velocity on validated growth charts [11]. Poor weight gain, low BMI, and/or slowed linear growth velocity may all suggest poor disease control and increased risk for delayed puberty. Regular physical examination of pubertal status, primarily assessing for appropriate breast development, is important to assess for any stalling or regression of pubertal development [4]. A bone age radiograph and 8AM serum estradiol, FSH, and LH levels using ultrasensitive (i.e., pediatric) assays are useful screening tests to obtain when physical exam findings are equivocal (see Table 1) [3]. 

Once delayed puberty has been identified in a female with IBD, optimizing nutrition and treatment of IBD are essential to allow for normal pubertal progression [4, 5, 12]. Suspicion of alternative etiologies for pubertal delay warrants prompt referral to pediatric endocrinology and coordination with the patient’s primary care provider to facilitate other subspecialty referrals as needed. For instance, a 13-year-old female with advanced pubic hair development, but no breast development should undergo evaluation for estrogen deficiency of gonadal or hypothalamic origin. In general, any female with delayed puberty and well controlled IBD and/or concern for impaired bone mass accrual should be evaluated by an endocrinologist [4]. 

## Menstruation and Menstrual Cycle Disorders

### Menstrual Cycle Disorders in Patients with IBD

Primary dysmenorrhea, or painful menstrual cramps without underlying pathology, is the most common gynecological complaint among women of reproductive age [13], with prevalence estimates ranging from 17 to 81% [14]. Primary dysmenorrhea occurs at least as frequently in the IBD population, affecting approximately 40–62% of women [15, 16]. In addition, active IBD can lead to irregular cycles, menorrhagia (excessively heavy menses), and hormone-sensitive gastrointestinal symptoms [17, 18]. In some cases, changes in menstrual cycles may even precede a diagnosis of IBD; 30% of surveyed women reported changes in their menstrual cycle interval in the year preceding their IBD diagnosis, and among those with baseline dysmenorrhea, 33% reported increased pain intensity [19]. In addition, endometriosis, a condition characterized by endometrial tissue located outside of the uterus, is a cause of secondary dysmenorrhea and can mimic abdominopelvic pain as experienced in patients with IBD [20]. 

### Relationship between IBD and Menstrual Symptoms

Many women with IBD notice cyclical changes in their GI symptoms around the time of menses, including increases in abdominopelvic pain, diarrhea, bloating, nausea, and constipation [18, 21, 22]. In one study, compared to healthy controls, women with IBD and irritable bowel syndrome were more likely to experience cyclical changes in bowel habits associated with menses [23]. In another study, women with IBD in remission and healthy controls were both found to have a higher frequency of defecation during menstruation than in between cycles, although women with active CD did not report cyclical variation in GI symptoms [24]. Prevailing theories to explain worsened GI symptoms during menses include increased endometrial prostaglandin release leading to increased intestinal inflammation and motility and/or activation of estrogen receptors in the GI tract triggering visceral hypersensitivity and increased motility [18, 21, 25]. Accordingly, cyclical changes related to menses can be mistaken for IBD symptoms and lead to unnecessary escalation of IBD treatment [15, 18]. While dysmenorrhea-associated symptoms and active IBD symptoms often overlap, certain features can help distinguish between them; menorrhagia and breast tenderness align more closely with dysmenorrhea, while gastrointestinal bleeding, nocturnal stools, or weight loss point to an IBD flare [18, 19]. If a patient presents with increased symptoms at the time of their menstrual period, providers should evaluate for inflammation with objective biomarkers and address any menstrual symptoms before considering IBD treatment changes [18]. 

### Impact of Menstrual Cycle Disorders on Functioning and Quality of Life

Menstrual cycle disorders can significantly impair daily functioning and quality of life [13, 26], and this phenomenon may be compounded for women with IBD [15, 18]. A cross-sectional study of female adolescents found that 38% have missed school due to menstrual pain [27]. Menstrual symptoms may also interfere with daily activities, impair work performance, and lead to absenteeism [14, 28, 29]. In a study of > 1,000 women with IBD, those with worsening GI symptoms at the time of menses had lower quality of life (QOL) scores than those without hormonally-sensitive symptoms [30]. Similarly, in a cohort of women with CD, those who reported dysmenorrhea had significantly lower health-related QOL than those without (*p* = 0.016) [15]. 

### Managing Menstrual Cycle Disorders in Women with IBD

The American College of Obstetrics and Gynecology recommends using non-steroidal anti-inflammatory drugs (NSAIDs) and/or hormonal suppression as first-line for treating primary dysmenorrhea and menorrhagia [31], but both treatments may have limitations for women with IBD. NSAIDs effectively ameliorate dysmenorrhea by blocking prostaglandin-mediated uterine smooth muscle contractions [23], and trials suggest that NSAIDs treat menstrual pain better than alternatives, including acetaminophen [14, 32–37]. However, NSAIDs, particularly when taken at high doses or for prolonged periods, can exacerbate IBD [38–41]. Importantly, this finding is based on studies that (1) exposed patients to NSAIDs daily for weeks instead of the < 5-day duration typical for period pain or (2) were subject to biases such as confounding due to reverse causality [38, 40]. Accordingly, while providers may consider condoning occasional NSAIDs for their patients with IBD suffering with menstrual cycle disorders, additional randomized trial data is needed to guide decision-making.

Hormonal contraception, on the other hand, prevents endometrial proliferation and ovulation, decreasing menstrual bleeding and mitigating uterine cramps by downregulating prostaglandin and leukotriene production [31]. See the *Contraception* section for details on using hormonal contraception for menstrual cycle disorders in IBD. Non-pharmacologic therapies merit consideration as well, particularly topical heat application and exercise [31]. Other complementary treatments, including yoga, acupuncture, and transcutaneous electrical nerve stimulation, have shown benefit in some studies, but data remain limited [31]. 

For women with menorrhagia and IBD, providers should assess for and treat iron deficiency anemia, as both conditions can deplete iron stores [42]. Inflammation leads to upregulation of hepcidin, an iron regulatory hormone that decreases duodenal iron absorption and limits utilization of stored body iron [42]. As a result, oral iron may inadequately replete iron stores in patients with active inflammation, and intravenous iron should be considered [42, 43]. 

## Contraception

Pediatric gastroenterologists are often asked about the safety of contraceptive methods. These discussions are important, as adolescents and young women with IBD are at risk for unintended pregnancy which can be associated with poor maternal and fetal outcomes in the setting of poorly controlled IBD [44–46]. In addition, contraceptives are also indicated for a variety of other conditions including menstrual cycle disorders, polycystic ovary syndrome, and acne [15–18]. A complete contraceptive conversation should include discussions about privacy, ease of starting/stopping a method, side effects, impact on uterine bleeding, effectiveness at pregnancy prevention, and rate of return of fertility after discontinuing a method [47]. While many of these items may be addressed by a primary care or reproductive health provider, studies repeatedly demonstrate that reproductive-age women with IBD desire discussions about SRH, including contraception, with their GI providers [22, 48, 49]. The pediatric gastroenterologist should have an understanding of contraceptive options and IBD-specific considerations.

It is important to note that there are no contraceptive methods that are absolutely contraindicated in adolescents and young women with IBD, nor any known drug-drug interactions between contraceptives and IBD therapies [44, 50]. Below, we will describe each method, its effectiveness in preventing pregnancy, and any IBD-specific considerations.

### Barrier Methods

Barrier methods include internal or external condoms, diaphragms, cervical caps, sponges, spermicides, and vaginal pH modulators. These methods have high typical use failure rates ranging from 13 to 27% [51]. While safe for use in women with IBD, they do not have any additional health benefits beyond pregnancy prevention and aside from condoms, do not protect against sexually transmitted infections.

### Hormonal Methods

Hormonal contraceptives are divided into two categories: progestin-only versus combined hormonal contraceptives (CHCs) which contain both estrogen and progesterone. Progestin-only methods come in the form of progesterone-only pills, subcutaneous depo-medroxyprogesterone (DMPA) injections, etonogestrel subdermal implants, and levonorgestrel-containing intrauterine devices (LNG-IUD). The latter 2 agents are referred to collectively as long acting reversible contraceptives (LARCs). CHCs come in the form of combined oral contraceptive pills (OCPs), transdermal patches, or vaginal rings [51]. Typical use failure rate for hormonal methods ranges from 4 to 7% with the exception of LARCs which have a failure rate of < 1%. In addition to pregnancy prevention, hormonal contraceptives are used to treat menstrual cycle disorders (dysmenorrhea, menorrhagia, endometriosis), premenstrual mood disorders, and acne [31, 52, 53]. 

### IBD Specific Contraceptive Considerations

One of the greatest concerns related to contraception in women with IBD is venous thromboembolism (VTE) risk in those receiving CHCs, which is significantly elevated both amongst those with IBD as well as those on CHCs [54, 55]. Compared to controls, women with IBD have a 2- to 3-fold increased risk of VTE, increasing up to 8-fold in the setting of active IBD [56, 57]. To date, only one study compared VTE rate among women with IBD on OCPs versus those without; while this study found no significant difference, it was limited by retrospective trial design and small numbers [58, 59]. Leading GI organizations advocate for a careful assessment of thrombotic risk before prescribing CHCs to patients with IBD, and recommend LARCs as first-line options in IBD given effectiveness in pregnancy prevention, menstrual cycle disorder management, and lack of VTE risk [60, 61]. However, the Center for Disease Control’s US Medical Eligibility Criteria for Contraceptive Use advises that the benefits of CHCs generally outweigh the risks for women who have IBD but no other thromboembolic risk factors (extensive active disease, recent surgery, immobilization, smoking, obesity, or fluid depletion) [62]. Accordingly, in a woman with mild or inactive disease without risk factors, the benefits of CHCs in terms of pregnancy prevention (during which VTE risk is increased 5-fold) and ease/acceptability of use may outweigh the risks [63]. Of note, CHCs remain the most popular type of prescription contraceptive in IBD, making up 72% of contraceptives amongst US women ages 15–25 compared to < 2% LARCs [49, 64]. 

CHCs have also been questioned as a potential risk factor for IBD relapse, but data are conflicting and overall of low quality. A single prospective cohort study reported a roughly 3-fold higher risk of disease activity among CHC users compared with non-users; however, this was limited to untreated CD and CHC users were younger with a younger age at diagnosis—both independent risk factors for relapse—and several other prospective cohorts and a recent systematic review did not confirm this association [50, 65–67]. 

A concern relevant to all oral contraceptive methods relates to absorption; since oral contraceptive pills (OCPs) are absorbed in the small bowel, there is a theoretical risk of malabsorption in a patient with extensive small bowel disease or resection. However, in studies comparing women with UC with and without colectomy, and women with CD with and without limited ileal resection, there were no significant differences in plasma drug concentration [68, 69]. 

When considering DMPA injections, patients and clinicians should be aware of the association with reversible bone loss, which may compound low bone mineral density associated with IBD [70]. While no specific recommendations exist regarding monitoring or treatment of bone mineral density in women with IBD on DMPA, densitometry surveillance should be pursued per national guidelines [71], and providers can consider earlier assessment in patients with multiple risk factors like severe malnutrition, amenorrhea, or chronic corticosteroid exposure.

Finally, there are a few considerations regarding LARCs in women with IBD. First, while highly effective at pregnancy prevention, the copper IUD may cause cramping and heavy, irregular bleeding [72]. Second, while implant and IUD placement procedures are typically done in the office without anesthesia or sedation, younger patients or those with procedural anxiety or a trauma history may benefit from local anesthetics, sedated procedures, or coordination with other sedated GI procedures or imaging [73]. 

When discussing contraceptive methods with young women with IBD, providers should take a patient-centered, shared decision-making approach [47, 74]. The pediatric GI clinician should integrate disease-specific medical information with the patient’s goals, values, and preferences, allowing them to weight risks and benefits and make informed choices.

### Fertility and Pregnancy

Due to a complex interplay of disease-specific factors, post-surgical changes, and psychosocial factors, having IBD impacts fertility and pregnancy outcomes. Despite guidelines designed to optimize these outcomes, misperceptions and misinformation among patients and providers persist. It is critical that views surrounding fertility and pregnancy are informed by evidence to reduce unnecessary harm to patients and to protect their reproductive choice. Given the chronicity of IBD, while the majority of patients cared for in the pediatric setting are not imminently considering pregnancy, early discussions of the impact of IBD on fertility, the value of tight control of inflammatory disease in regard to maternal and fetal outcomes, and the safety of the vast majority of IBD therapies during pregnancy and lactation is essential.

### Fertility and Childbearing in Women with IBD

Fertility rates in women with IBD in remission with no prior pelvic surgery approximate those in the general population [60]. However, those with active or relapsing disease, a history of ileal-pouch anal anastomosis (IPAA), or proctectomy and permanent ostomy have decreased rates, likely due to decreased ovarian reserve and tubal factor infertility from pelvic inflammation and adhesive disease [60, 75]. IPAA has been shown to have as high as 40–54% reduction in fertility in the 6–12 months following surgery, though rates improve over time and may be more favorable with a laparoscopic versus open surgical approach [76, 77]. Conversely, ileorectal anastomosis (IRA) alone has not been shown to decrease fertility, though IBD outcomes may be subpar [75]. Ultimately, IBD surgical decisions are nuanced and must balance the risks and benefits of varied surgical approaches, IBD outcomes, and impacts on fertility. Providers should tailor counseling regarding fertility based on patients’ surgical histories and current disease activity. For those with complex surgical histories and those who do not achieve spontaneous pregnancy within 6 months, referral should be made to a fertility specialist [60]. Importantly, medical therapy for control of IBD does not decrease female fertility [76, 78]. 

Up to 17% of women with IBD report voluntary childlessness [79]. While active disease may play a role in this decision (women with IBD without children report higher rates of hospital admissions and surgical interventions), many women elect not to have children due to concerns surrounding heritability, risk of congenital anomalies, peripartum active disease, and teratogenicity of medications [80]. There is a 5-10-fold increased risk of developing IBD among children of parents with IBD, but the absolute risk remains relatively low, between 5 and 13% [81, 82]. Evidence-based education improves patient knowledge, minimizes misconceptions, and allows patients to make more informed decisions about childbearing [79, 83]. 

### Pregnancy Considerations and Preconception Counseling

The Global Consensus Statement regarding the management of pregnancy in IBD recommend that preconception counseling begins at least 6 months prior to attempting conception [44]. Once pregnancy is achieved, uncontrolled disease poses the greatest threat to both mother and fetus, with risks including spontaneous abortion, low birth weight, intrauterine growth restriction, preterm labor, preterm premature rupture of membranes, inadequate maternal gestational weight gain, and maternal preeclampsia [76]. Remission at conception optimizes pregnancy disease course and outcomes. Preconception counseling helps patients to achieve adequate disease control [76, 84, 85] and address modifiable risk factors including smoking cessation [86]. 

Nearly all standard medications (aminosalicylates, biologics, and 6-mercaptopurine) may be continued through pregnancy and delivery to achieve disease control [60]. Patients should stop methotrexate at least three months prior to conception due to teratogenicity, and avoid long-term corticosteroid use due to adverse fetal effects. Small molecule therapies should be avoided in pregnancy when feasible, as animal models suggest teratogenicity at high doses [87]. Patients on sulfasalazine should receive 2 mg of folic acid daily to prevent neural tube defects. Additionally, patients may be counseled that with the exception of methotrexate and small molecule therapies, IBD therapies are also safe during lactation [61, 87]. 

The importance of nutrition to assure wellness as well as to potentially treat IBD is recognized [88]. While a dedicated discussion of the nutritional management of IBD is beyond the scope of this review, it is important to highlight that optimizing maternal diet in the the pre-conception and peripartum periods benefits both mother and fetus [44]. Notably, nutritional deficiencies (iron, vitamin B12, and folate) and malabsorption related to IBD can adversely impact fetal development. Registered dietician assessment and appropriate micro- and macronutrient supplementation can help patients meet safe intake requirements for pregnancy [60]. In addition, the prevention of IBD through diet and its effects on the gut microbiome represents an exciting future direction for the field [89]. 

### Preventive Care

Human papillomavirus (HPV) currently affects approximately 42 million US individuals, with 13 million new infections annually [90]. HPV causes more than 36,000 cases of cervical, penile, vulvar, vaginal, anal and oropharyngeal cancers in the U.S. annually. High-risk HPV types 16 and 18 cause ~ 70% of invasive cervical cancers, while types 31, 33, 45, 52, and 58 account for an additional ~ 20% [91]. Cervical cancer remains a major health concern in the U.S., with ~ 13,000 new cases and > 4,000 deaths annually [92]. Since the Food and Drug Administration (FDA) approved the first HPV vaccination in 2006, HPV incidence declined markedly, with population-based studies confirming reductions in precancerous lesions and invasive cervical cancer [92]. 

Adolescents with IBD are a priority population for cervical cancer prevention. Oral cancer occurs more frequently among individuals with IBD [93], and perianal disease further increases the likelihood of HPV-related anal malignancies [94]. Women with IBD on immunosuppressive medication show higher rates of abnormal Papanicolaou (Pap) test results than both patients with IBD not on immunosuppressive medications or the general population [95–98]. Thus, routine HPV vaccination and cervical screening are essential for reducing the risk of cervical cancer for patients with IBD.

### HPV Vaccination

Gardasil 9, the only Human Papilloma virus (HPV) vaccine currently used in the U.S., was FDA approved in 2014 [99]. It protects against HPV subtypes 6, 11, 16, 18, 31, 33, 45, 52, and 58 [100] Types 6 and 11 cause ~ 90% of genital warts, while the other high-risk types account for nearly all cervical cancers [91, 99]. The CDC and ACIP recommend routine vaccination at ages 11–12, though it may be initiated as early as age 9, and should ideally be completed by age 26 [99]. Two doses (at 0 and 6–12 months) are sufficient if completed before age 15; however, three doses (at 0, 1–2, and 6 months) are advised for those beginning the series at 15 or older. Additionally, all immunosuppressed patients, including those with IBD, should receive the three-dose series, even if they begin the series before the age of 15 [101]. Therefore, patients should be advised to receive the HPV vaccine series prior to initiating immunosuppression wherever possible.

Studies demonstrate the vaccine is safe and immunogenic in IBD [102], though antibody responses may be blunted under immunosuppression [103]. Barriers to HPV vaccination include missed opportunities in subspecialty clinics, safety-related misconceptions, and stigma of HPV linked to sexual activity [104–107]. Integrating vaccination into infusion centers or GI clinics may improve uptake [108]. IBD providers should advocate strongly for HPV vaccination in their patients.

### Cervical Cancer Screening

National guidelines recommend cervical cancer screening with Pap smear beginning at age 21 regardless of sexual activity [109, 110]. For women aged 21–29, cytology every three years is preferred; after age 30, options expand to include cytology alone, HPV testing alone or co-testing (i.e., HPV and cytology) [109]. These strategies reduce cancer incidence and mortality. National guidelines recommend cervical screening beginning at age 21 regardless of sexual activity [109, 110]. For women aged 21–29, cytology every three years is preferred; after age 30, options expand to include cytology alone, HPV testing alone or co-testing (i.e., HPV and cytology) [109]. These strategies have been found to reduce cancer incidence and mortality [111, 112]. 

While IBD patients without immunosuppression can follow routine guidelines, IBD patients on immunosuppression face heightened risk of cervical dysplasia [95–98]. While evidence does not mandate earlier initiation, shorter intervals may be considered for those on immunosuppression [113]. The American College of Gastroenterology recommends that cytology be performed for immunosuppressed individuals annually within a year of the onset of sexual activity; if three consecutive tests are negative, patients can extend screening intervals to every 3 years [114]. Unfortunately, barriers are common: young adults often lack access to gynecologic care, hesitate to undergo pelvic exams, and face fragmented responsibility between GI, primary care, and gynecology [115, 116]. Multidisciplinary collaboration and multimodal interventions are essential to ensure preventive care is not missed.

## Counseling Approaches

Proactive counseling in all clinical care settings is an essential aspect of pediatric IBD care, particularly for female adolescents managing both chronic illness and developmental transitions. Early, developmentally appropriate discussions about SRH support informed decision-making, foster autonomy, and promote overall well-being [117]. For adolescents with IBD, counseling must also consider the psychosocial impact of chronic disease, medication-related risks, and concerns about fertility and body image [118]. Fig. 1 provides a guide for asking about and addressing SRH topics with the young patients with IBD in mind, and Table 2 highlights high-yield patient-facing resources to support these conversations.

### Counseling Strategies

Counseling should be tailored to the adolescent’s cognitive and emotional maturity. For younger teens, the focus may be on puberty, growth, and how IBD affects menstruation. For older adolescents, counseling should include contraception, STI prevention, reproductive planning, and the teratogenic risks of certain medications [22, 60, 119]. Counseling strategies should be individualized according to disease activity, developmental stage, and patient values [120]. Special attention is warranted for those with prior surgeries that may affect fertility or those prescribed teratogenic drugs. Providers should introduce sensitive topics gradually and with respect for cultural and family context, building trust and allowing for ongoing dialogue [121]. Providing confidential, one-on-one time with the patient builds trust, encourages disclosure of sensitive concerns, and ensures adolescents understand confidentiality boundaries [117]. While parents remain critical caregivers, particularly in IBD management, clinicians must balance family involvement with the adolescent’s emerging legal right to minor consent and developmental need for autonomy in SRH decisions [122]. 

However, significant barriers to high quality counseling exist. Gastroenterologists may feel discomfort discussing sexual health, face time constraints in GI visits, or lack training in adolescent-centered communication [123]. Patients may perceive stigma or encounter fragmented care between specialties. Strategies to address these gaps may include brief screening tools to assess sexual health needs, professional training in adolescent sexual health communication, and delegation of preventive counseling to nurses or health educators [124]. Creating a supportive clinic environment—through visible educational materials and clear policies on confidential services—can normalize discussions and encourage adolescent engagement [125]. Moreover, implementing standardized training in IBD-specific SRH for pediatric gastroenterologists would likely enhance SRH care delivery, given that structured education has meaningfully improved outcomes in other subspecialties in which chronic conditions impact SRH, such as cardio-obstetrics [126]. 

## Conclusions

IBD impacts adolescents and young women during their prime developmental and reproductive years. To provide optimal care for these patients, pediatric GI providers must understand how IBD impacts SRH. By screening for and managing SRH-related concerns, IBD providers can significantly improve patient QOL and overall outcomes. While discussions of fertility and pregnancy seem far off for many in the pediatric setting, patients and families often have questions about these topics and pediatric providers can educate and empower patients to understand their health and make decisions to support their future.

## Key References

- Nguyen AT, Curtis KM, Tepper NK, Kortsmit K, Brittain AW, Snyder EM, et al. U.S. Medical Eligibility Criteria for Contraceptive Use, 2024. MMWR Recommendations and reports : Morbidity and mortality weekly report Recommendations and reports. 2024;73(4):1–126.
  - ○ Reviews recommendations for contraceptive use taking into consideration medical conditions, including inflammatory bowel diseases.
- Ott MA, Sucato GS, Leroy-Melamed M, Hoopes AJ, Adolescence Co. Contraceptive Counseling and Methods for Adolescents: Clinical Report. Pediatrics. 2025;156(1).
  - ○ Developed for the pediatric provider, this report provides information about adolescent-centered counseling approaches and contraceptive methods.
- Mahadevan U, Seow CH, Barnes EL, Chaparro M, Flanagan E, Friedman S, et al. Global Consensus Statement on the Management of Pregnancy in Inflammatory Bowel Disease. Journal of Crohn's and Colitis. 2025;19(8).
  - ○ This consensus statement provided evidence-based, practical guidelines to care for pregnant patients with IBD including pre-conception counseling, disease management during pregnancy, and post-delivery considerations for offspring.
- Farraye FA, Melmed GY, Lichtenstein GR, Barnes EL, Limketkai BN, Caldera F, et al. ACG Clinical Guideline Update: Preventive Care in Inflammatory Bowel Disease. Am J Gastroenterol. 2025;120(7):1447–73.
  - ○ Clinical guideline that reviews preventive care recommendations for patients with IBD, including but not limited to information about HPV vaccination and cervical cancer screening.
- Allison BA, Wilkinson TA, Maslowsky J. Adolescent-Centered Sexual and Reproductive Health Communication. Jama. 2025;333(3):250–1.
  - ○ This manuscript reviews effective methods for the provision of person-centered SRH care provision.

## Data Availability

No datasets were generated or analyzed during the current study.
